# Prevention of Autogenous Shrinkage in High-Strength Mortars with Saturated Tea Waste Particles

**DOI:** 10.3390/ma12172654

**Published:** 2019-08-21

**Authors:** Sadam Hussain Jakhrani, Jae Suk Ryou, Atta-ur-Rehman, In Kyu Jeon, Byeong Hun Woo, Hong Gi Kim

**Affiliations:** Department of Civil and Environmental Engineering, Hanyang University, Seoul 04763, Korea

**Keywords:** black tea waste, flow, autogenous shrinkage, high-strength mortar, microstructure

## Abstract

The purpose of this study was to prevent early age autogenous shrinkage in high-strength mortars with saturated tea waste particles. In general, high strength and high performance concretes are made with low water/binder ratios; hence, they are susceptible to shrink at early ages. This shrinkage occurs due to self-desiccation that leads to autogenous shrinkage. To overcome self-desiccation problems in high-strength cement composites, it is necessary to keep the composites moist for a long time. Pre-saturated porous lightweight aggregates and super absorbent polymers are the most commonly used materials in high-strength cement composites to keep them moist for a long time; however, in this study, porous tea waste particles were used to keep the cement mortars moist. Pre-saturated tea waste particles were used in two different size proportions, making up as much as 3% of the volume of the binder. Moreover, commonly used lightweight aggregate (perlite) was also used to compare the outcomes of specimens made with tea waste particles. Different parameters were observed, such as, flow of fresh mortars, autogenous shrinkage, mechanical strengths and microstructure of specimens. The addition of tea waste and perlite particles in mortars made with Ordinary Portland cement (OPC) as the only binder, showed a reduction in flow, autogenous shrinkage and mechanical strengths, as compared to mixes made with partial addition of silica fume. Although, the use of silica fume improved the mechanical strength of specimens. Moreover, the use of saturated tea waste and perlite particles also improved the microstructure of specimens at an age of 28 days. The results revealed that the saturated tea waste particles have the ability to prevent autogenous shrinkage but they reduce strength of high-strength mortars at early ages.

## 1. Introduction

High-strength concretes (HSCs) and ultrahigh performance concretes (UHPCs) are the most commonly used concretes in today’s construction industry due to their excellent ductility, durability and strengths [1]. However, they are susceptible to early age shrinkage known as autogenous shrinkage due to self-desiccation [2,3,4]. According to Dudziak and Mechtcherine [5], early age shrinkage leads to micro cracking and then shows adverse effects on durability and strength properties in HSCs and UHPCs. Aitcin et al. [6] anticipated that the autogenous shrinkage in high-performance concretes occurs due to rapid self-desiccation, and found that this was due to low water/binder (w/b) ratios. Lura et al. [7] reported that the autogenous shrinkage in high-strength concretes is due to loss of interior moisture in cement composites which results in high internal stresses and cracking. Bentz and Snyder, and Boshoff and Combrinck [8,9] discovered that the hydration of cement leaves empty pores within the cement paste, which reduces the internal relative humidity, leading to self-desiccation. Jiang et al. and Li et al. [10,11] observed that the reduction of internal relative humidity during hydration in cement mix is the major source of early age shrinkage in HSCs and UHPCs.

To overcome the problem of shrinkage in Ordinary Portland cement (OPC)-based composites, two common methods have been suggested so far (i.e., using chemical-based shrinkage reducing admixtures (SRAs) and the internal curing (IC) method [12]). The use of SRAs is a commonly adopted method to reduce long-term shrinkage, especially drying shrinkage; however, due to their high costs and lack of major effects on autogenous shrinkage during the hydration process, they are not recommended to be used for the said purpose [13,14,15]. On the other hand, IC is a cheap technique because of the availability of most of the materials/agents. Awareness about the usage of internal curing materials/agents (ICAs) was given by [16]. It was suggested that the best ICAs are those that exhibit humidity very close to 100% in cement composites and have an ability to carry water to other parts of the composite. Porous lightweight aggregates (PLWAs), super absorbent polymers (SAPs), superfine powders (SFPs) and other porous materials are recommended to be used in pre-saturated states to increase the internal relative humidity of such composites [16,17,18,19,20,21,22,23,24]. However, it was observed that these types of ICAs had issues of low absorption rates; low water-release rates during the hydration process and pore sizes smaller than the hardened pastes, which influence the proper supply of internal water to other parts [16,19,20,21,22].

Moreover, agro-based materials are also used as internal curing agents. Lura et al. [25] used biomass-derived waste material and considered it as a lightweight aggregate. Jakhrani et al. [26] used saturated black tea waste and perlite particles as internal curing agents to control rapid hydration in high-strength mortars. The use of agro-based waste material as building material was also suggested by [27]. They documented that the generation of agricultural waste has an adverse impact on the environment and recommended using such wastes to substitute conventional building materials to conserve traditional building materials and preserve the environment. Kadir and Masoom [28] used sugarcane bagasse waste as low thermal conductive material in fired clay bricks. Gorhan and Simsek [29] added rice husk into clay bricks to increase their porosity. Munoz et al. [30] added pomace waste for making clay bricks and observed no adverse impact up to the addition of 5%, however, an addition more than 5% reduced the mechanical properties. Ozturk et al. [31] used tea waste at different amounts for making brick clay mixtures to study the influence of tea waste on the properties of fired brick. They established that the usage of tea waste up to 10% has no negative impact on structural properties, and suggested that tea waste can be used as pore-making agents in the production of bricks. Furthermore, the literature shows that the worldwide tea consumption has increased to about 5 million tons/year [32]. In another report, worldwide tea production was reported to increase about 4.4% over the decade following 2007, reaching 5.77 million metric tons in 2016 [33]. As perceived from the reviewed literature, huge amounts of tea waste are being produced and thrown in the open environment, making it dirty and polluted. Therefore, it is necessary to utilize this waste to make the environment green and get rid of such agro-based wastes. In addition, a few researchers approached the utilization of tea waste as building materials. Therefore, to broaden the utilization of tea waste as construction and building material, this work was performed.

This study uses agro-based waste material (black tea waste) in an effort to minimize its adverse environmental impacts. However, the high absorption ability of black tea waste makes it an appropriate internal curing agent to be used for internal curing purposes to prevent early age autogenous shrinkage in high-strength mortars to avoid propagation of leading cracks due to high shrinkages.

## 2. Experimental Process

### 2.1. Materials

Mortar specimens are made with different materials. OPC under the ASIA brand was purchased from a local market in Seoul, Korea. OPC conformed to ASTM C150 [34] with a specific gravity of 3.15 g/cm^3^ and Blaine fineness of 325 m^2^/kg. Silica fume (SF) was purchased from a local Korean company named Chemicon, Gyongi-do, Korea, with a specific gravity of 2.2 g/cm^3^ and Blaine fineness of 20,000 m^2^/kg. Both OPC and silica fume were used as binders. The detailed chemical composition and physical properties of used binders are given in Table 1. Natural river sand with a specific gravity of 2.65 g/cm^3^, fineness modulus of 2.7, absorption of 1.05% and maximum particle size of 4.75 mm was used as fine aggregate. Black tea waste (TW) and perlite (P) particles were used as internal curing agents. TW particles were collected from foreign students’ cafes and local restaurants in domestic areas located in Seoul, while the perlite particles were purchased from a local Korean-based dealer; however, the actual brand was from a Chinese company, named Shijiazhuang Kedahua Imp-Exp Trade Co. Ltd. (Shijiazhuang, China). Two different size ranges of black TW and perlite particles were used in various mortar mixes. The two different particle sizes were numbered TW30, TW50, P30 and P50. The number 30 shows that the black TW or perlite particles passed through ASTM sieve No. 4 and were retained on sieve No. 30, whereas, the number 50 shows that the particles passed through ASTM sieve No. 30 and were retained on sieve No. 50. The observed physical properties of two different types of tea waste and perlite particles are given in Table 2, however, the observed elemental composition of used black tea waste is given in Table 3. Poly-carboxylate liquid-type, high range water reducing admixture conforming to ASTM C494 [35] was used as a super plasticizer (S.P) to improve the workability of mortars.

### 2.2. Mix Proportions

In this study, a total of 18 mixes were made with the acronyms given as control mix, OPC/TW30-1, OPC/TW30-3, OPC/TW50-1, OPC/TW50-3, OPC/P30-1, OPC/P30-3, OPC/P50-1, OPC/P50-3, SF-10, SF-10/TW30-1, SF-10/TW30-3, SF-10/TW50-1, SF-10/TW50-3, SF-10/P30-1, SF-10/P30-3, SF-10/P50-1 and SF-10/P50-3. Mortar mixes starting with the acronym OPC indicate that the mixes are made with OPC as the only binder, while the mixes beginning with SF represent the mixes that are made with a fractional replacement of OPC with silica fume. The mixes, control mix and SF-10, did not contain tea waste and perlite particles, however, the remaining 16 mixes contained two different sizes of tea waste or perlite particles, depending upon the nature of the made mix. In addition, the numbers 1 and 3 at the end of the mixes show the amount of added tea waste or perlite particles by volume of binders, and the number 10 in the mixes indicates the replacement of OPC with 10% silica fume by weight. For all mixes, total water/binder (w/b) ratio = 0.20 is adjusted as mixing water; however, some part of this water is used for pre-saturating tea waste and perlite particles. Super plasticizer/binder (s.p/b) ratio = 0.01 is adjusted in all mixes for each test, except the autogenous shrinkage test, and in that case (s.p/b) = 0.02 is used to place fresh mortar easily in corrugated plastic tubes. The detailed mix proportion is given in Table 4.

### 2.3. Mixing

Before making the mortar specimens for all 18 mixes, all mortar ingredients are batched in the laboratory in dry or liquid form, depending on the nature of materials. Mixing for each mix is performed separately, using a small mixer with a capacity of 5 L. The mixing method for the first 9 mixes was similar as followed in our previous research work [26]. The difference in mixing of the remaining 9 mixes as compared to the previously made 9 mixes was the partial replacement of OPC with SF. In the case of mix SF-10, OPC and SF were mixed together with saturated surface dry (SSD) sand for 1 minute (min), then water and super plasticizer were added and mixed for an additional two min, resulting in a total mixing time of 3 min. For the remaining 8 mixes (SF-10/TW30-1, SF-10/TW30-3, SF-10/TW50-1, SF-10/TW50-3, SF-10/P30-1, SF-10/P30-3, SF-10/P50-1, SF-10/P50-3), the OPC and SF were first mixed for 1 min in dry form. Then, SSD sand and fully saturated tea waste or perlite particles (depending upon the type of mix) were placed in the mixer for an additional 2 min. Finally, the water and super plasticizer were added, and mixed for an additional 2 min, resulting in a total mixing time of 5 min for each mix. The mixing time increased from 3 to 5 min to ensure a proper homogenous mix. After the mixing process, the molds for making cubic specimens or prismatic specimens or other tests were thoroughly cleaned and/or oiled to remove dust from them. Then, fresh mortar was put into the respective molds for the designated period. Before putting fresh mortar into the molds, some mortar was removed from the fresh mix for the mortar flow test, and then the remaining mortar was placed in different molds for other investigations.

It should be noted that, before using tea waste particles in mixes, they were washed with potable clean water for 2 h to remove impurities like milk, sugars and other impurities that were absorbed/adsorbed by tea waste particles. After washing in clean water, the particles were boiled in hot water for 1 h to remove further impurities like coloring agents, etc. The cleaned and boiled tea waste particles were dried in open air in the sunlight for an entire day, and then were dried in the oven for 24 h at a temperature of 60 °C to make them purely dry. Finally, these cleaned and dried tea waste particles were brought into a fully saturated state before adding in mix.

### 2.4. Tests on Fresh and Hardened Mortars

#### 2.4.1. Flow Table Test

Flow of fresh mortars was measured using the flow table according to ASTM C230 [36], and the specified procedure for measuring flow according to ASTM C1437 [37]. 

#### 2.4.2. Autogenous Shrinkage

ASTM C1698 [38] was followed to measure the autogenous shrinkage of mortars with high flow ability. Fresh mortar was placed in vertically handled corrugated plastic tubes with one side sealed with a sealing cap before mortar was placed inside. The tube was held straight vertically and was placed on a vibrator for ensuring proper compaction to remove air bubbles. After filling the tube, the other side was sealed with the sealing cap. The corrugated plastic tubes were then placed horizontally on a designated bench for the test as suggested in [38]. Two replicated specimens were used for each mix and their average is given in the Section 3. The displacement transducers were attached from one side of each mold, and the other side was attached with the data logger to record the displacement (expansion or contraction) automatically in a consecutive manner for 7 days with 5 min intervals, at room temperature of 23 ± 2 °C. 

#### 2.4.3. Compressive Strength

Compressive strength for mortar specimens was performed in accordance with ASTM C109 [39]. Universal testing machine (UTM) with model (CCM 200-A) from Shimadzu Corporation, Kyoto, Japan was used for the compressive strength test evaluation. The maximum load cell of the machine was 200 tons with a loading speed of 30 mm/min. Cubic mortar specimens 50 × 50 × 50 mm^3^ in size were prepared. Six cubic specimens were cast for each mix; among them, 3 replicate specimens were investigated after curing times of 7 and 28 days, respectively.

#### 2.4.4. Flexural Strength

The flexural strength test was executed in accordance with ASTM C348 [40]. The three-point bending machine with model (AG-1) from Shimadzu Corporation, Japan, with maximum loading capacity of 250 kN was used for flexural strength analysis. Prismatic mortar specimens 40 × 40 × 160 mm^3^ in size were prepared. Six prismatic specimens were cast for each mix. Among these, 3 replicate specimens were investigated after curing times of 7 and 28 days, respectively. 

#### 2.4.5. Ultrasonic Pulse Velocity (UPV)

The UPV test was done in accordance with ASTM C597 [41]. The test equipment (Ultra-con-170) from STANLAY^TM^ Company, New Delhi, India was used for evaluation of the UPV test. Prismatic mortar specimens 40 × 40 × 160 mm^3^ in size were prepared for this test evaluation. Six prismatic specimens were cast for each mix; among these, 3 replicate specimens were investigated after curing times of 7 and 28 days, respectively. 

#### 2.4.6. Densities and Absorption Rate of Hardened Mortar Specimens

Densities and absorption rates of hardened mortar specimens cured in water for 7 and 28 days were measured by the oven dry method. After the designated curing periods, specimens were taken out of water, then they were brought into saturated surface dry (SSD) condition before weighing. After weighing, they were put in a drying oven at a temperature of 105 ± 5 °C for 24 h. After 24 h, they were taken out of the oven, and were weighed again. In this way their densities and absorption abilities were checked. The absorption ability was measured using Equation (1), given in [26]:

(1) $$Absorption\left(\%\right)=\left(\frac{W_{SSD}-W_{OD}}{W_{OD}}\right)\times 100Absorption\left(\%\right)=\left(\frac{W_{SSD}-W_{OD}}{W_{OD}}\right)\times 100$$

#### 2.4.7. Scanning Electron Microscopy (SEM)

For SEM analysis, only 6 specimens were analyzed, depending upon their compressive strengths after 28 days curing. Samples 1 cm × 1 cm in size were cut from the middle portion of broken cubic specimens. After getting the required size of the samples, these were immersed in epoxy resin, and were allowed to harden in a vacuum desiccator for 24 h. After hardening, the surface was polished to create a smooth surface for final SEM analysis. SEM analysis was done to find the effect of internal curing with tea waste and perlite particles as compared to the control mix and the formed phases.

#### 2.4.8. Thermo-Gravimetric Analysis (TGA)

Perkin Elmer STA8000 instrument, available in the department of chemical engineering, Hanyang University, Seoul, Korea was used to analyze the weight loss of 6 mixed mortar specimens. The analysis was done in an N_2_ environment at a heating rate of 10 °C/min. The cubic mortar specimens that tested for compressive strength after 28 days were crushed into powder form by passing the powder through ASTM Sieve No. 200. The passed powder was used for TGA. TGA was done to recognize the weight loss of specimens made with tea waste and perlite particles as compared to the control mix.

## 3. Results and Discussion

### 3.1. Flow Table Test

**Figure 1:**
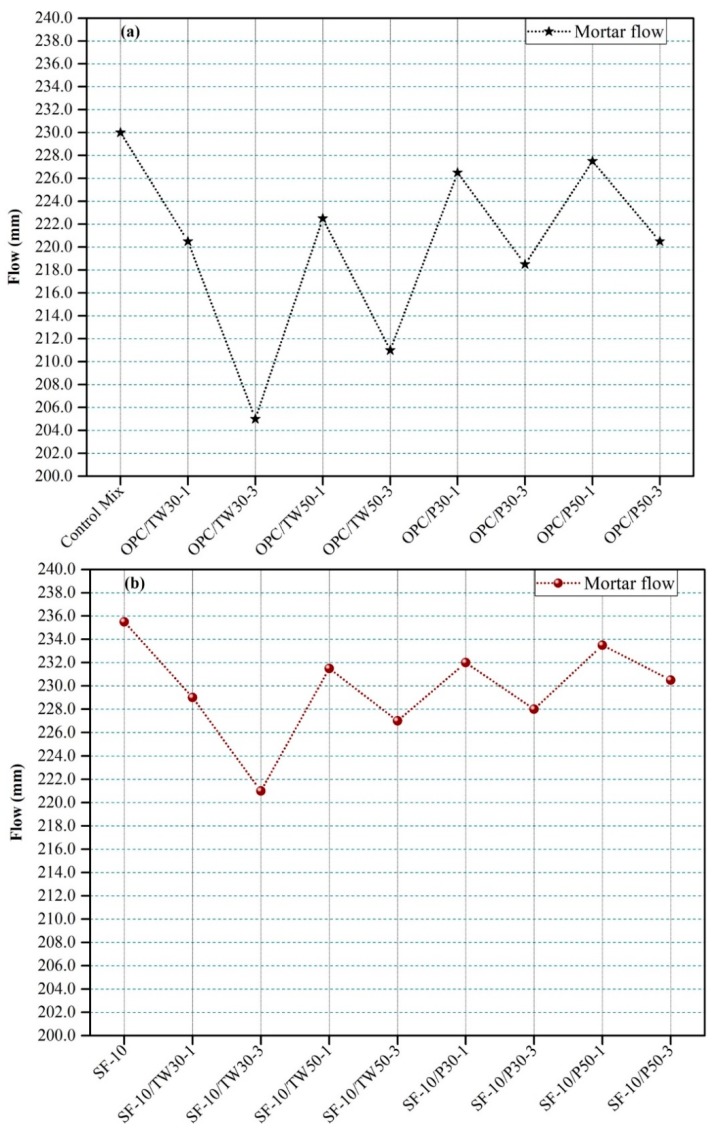
Flow of mortars made with tea waste and perlite particles. (**a**) 100% OPC and (**b**) 90% OPC and 10% SF.

The flow of fresh mortar of all 18 mixes was measured, and their averages are depicted in Figure 1. Figure 1a shows the flow of specimens made with OPC as the only binder, and Figure 1b demonstrates the average flow values of mixes made with partial replacement of OPC with silica fume. The control mix showed an average flow of 230.00 mm, which was high among the remaining 8 mixes that were made with tea waste or perlite particles with OPC as primary binder. The flow of mix SF-10 was 235.50 mm, which was high among the mixes that were made with tea waste or perlite particles with silica fume as the secondary binder. Very high and low flow values among all 18 mixes were obtained for mixes SF-10 and OPC/TW30-3, respectively. The results show that the addition of tea wastes and perlite particles cause reduction of flow. More reduction was observed in the mixes that contained coarser particles as compared to finer particles. Moreover, tea waste particles reduced more flow than perlite particles, which might be due to the high absorption ability of tea waste particles to not allow more flow to mortar as compared to perlite particles. However, the addition of silica fume slightly enhanced the flow as compared to the control mix and the respective mixes that were made with OPC as the only binder. 

The gained outcomes are coherent with the previous studies. Senff et al. [42] used different amounts of SAPs in mortars with different w/b ratios. They found a reduction in slump with increasing amounts of SAPs. Internal curing was done in UHPCs with SAPs by Justs et al. [43]. They found a reduction in the flow of concrete mixes that contained SAP particles. The slump flow was reduced to 770 mm from 1060 mm as compared to the control mix.

### 3.2. Autogenous Shrinkage

**Figure 2:**
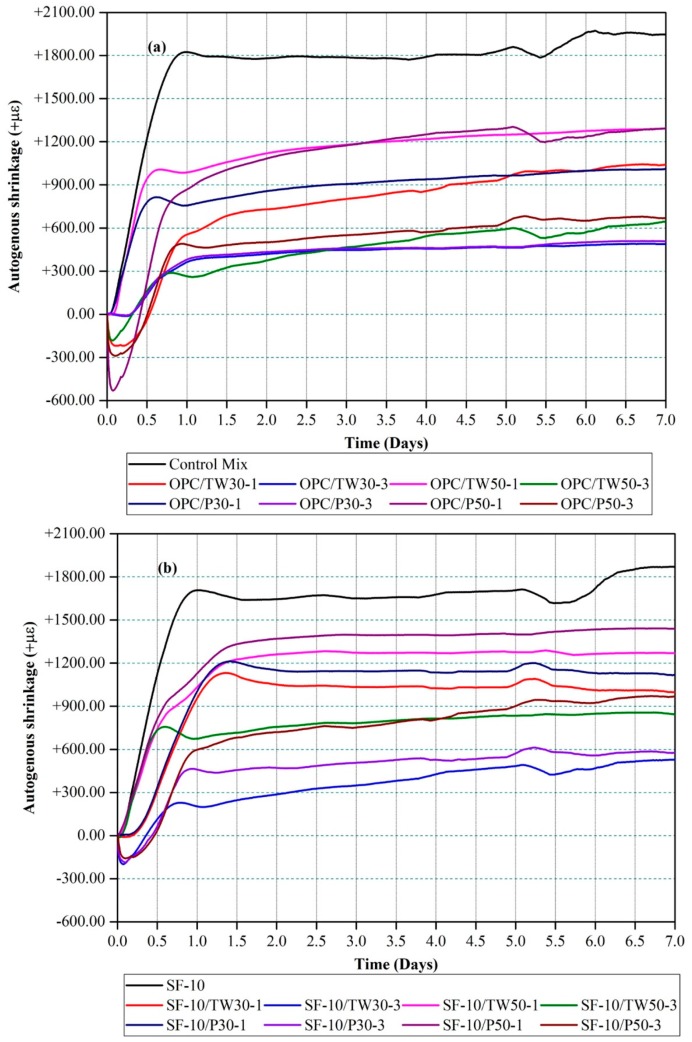
Autogenous shrinkage of mortars made with tea waste and perlite particles. (**a**) 100% OPC and (**b**) 90% OPC and 10% SF.

Autogenous shrinkage of all 18 mixes was measured, and their average values are given in micro strains (με) as shown in Figure 2. Figure 2a represents the shrinkage values of the first nine mixes, which were made with OPC as the only binder. The reference mix (control mix) was found to shrink rapidly after the first few hours, reaching shrinkage values of more than 1833.0 με at 1 day and 1952.5 με at 7 days. However, shrinkage values in the remaining eight mixes were found substantially decreasing. Among them, very low shrinkage values of 226.5 με and 531.3 με were obtained for the mix OPC/TW30-3 after 1 and 7 days, respectively. More reduction in shrinkage was observed for the mixes that contained a large amount of tea waste particles as compared to perlite particles. Figure 2b shows the shrinkage values of the remaining nine mixes that were partially made with silica fume. The addition of silica fume slightly increased the autogenous shrinkage for almost all mixes as compared to those without silica fume. The mix SF-10, which contained 10% silica fume and 90% OPC without tea waste and perlite particles showed shrinkage values of 1710.0 με and 1876.70 με at 1 and 7 days, respectively. Very low shrinkage values among the mixes made with SF were observed for mix SF-10/TW30-3 after 1 and 7 days. From the autogenous shrinkage results, it can be seen that the highest and lowest values were found for the control mix and OPC/TW30-3, respectively. It is concluded that the addition of certain amounts of porous pre-saturated tea waste and perlite particles can considerably reduce autogenous shrinkage.

The obtained consequences are comparable with the outcomes of previous studies. Zhou et al. [44] studied the effect of pore structure and absorption of lightweight aggregates (LWAs) on autogenous shrinkage. It was established that the pore structure and surface area of the LWAs influence the efficiency of shrinkage. Wang et al. [45] used recycled coral-based materials (RCBM) to assess the performance of ultrahigh performance concrete (UHPC), and observed that the addition of RCBM reduced autogenous early age shrinkage to some extent, and suggested that the porous nature of RCBM was the main reason. Liu et al. [46] used perforated cenospheres to replace 6% and 9% in mass of normal fine aggregates as an internal curing agent and found a substantial reduction in the autogenous shrinkage. Kumarappa et al. [47] made alkali-activated slag (AAS) mortars with and without LWAs. They obtained high autogenous shrinkage values for the mixes that did not contain LWAs and attributed this to the high contents of silica and Na_2_O in the mortars. Normal aggregates were then replaced with 20% saturated LWAs, which became more effective to control autogenous shrinkages. Oh et al. [48] used SAPs in AAS mortars and found a significant reduction of about 60–75% in autogenous shrinkage. Our research outcomes are also similar to findings from these researchers.

### 3.3. Compressive Strength

**Figure 3:**
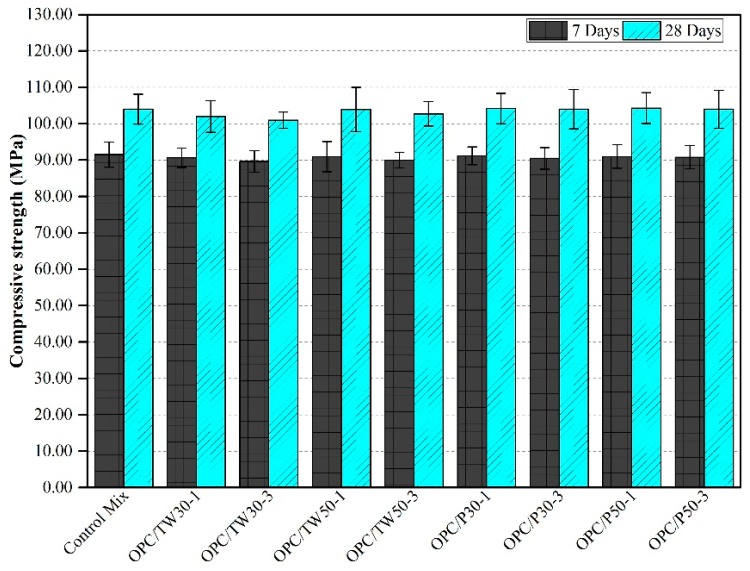
Compressive strength of mortars made with tea waste and perlite particles without SF.

**Figure 4:**
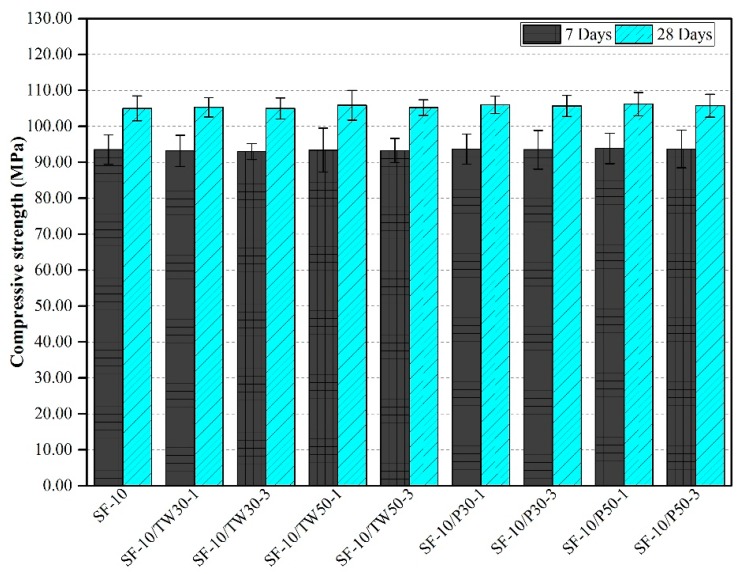
Compressive strength of mortars made with tea waste and perlite particles with SF.

The compressive strength results of all 18 mixes are shown in Figure 3 and Figure 4. Figure 3 indicates the compressive strengths of the first nine mixes that were made with only OPC as the binder. The average compressive strength of the control mix after 7 and 28 days was found to be 91.54 and 104.00 MPa, respectively. The compressive strength of mixes made with tea waste particles was slightly lower than that of the control mix, which was about 3.10% at 28 days, whereas, the specimen made with perlite particles also showed slightly lower strengths than the control mix, which was about 2.90% at 28 days. Overall, the strengths of specimens containing tea waste and perlite particles were lower than the control mix, where OPC was used as the only binder. Figure 4 represents the strengths of the remaining nine mixes that were made with partial substitution of OPC with silica fume. The addition of silica fume resulted in slightly enhanced compressive strengths of specimens as compared to those without silica fumes. The mix SF-10 gave a compressive strength of 93.50 and 105.98 MPa at 7 and 28 days, respectively. The usage of SF increased compressive strength about 1.86% in SF-10 as compared to the control mix. Similarly, the strength in SF-10/P-50-1 was 2.07% higher than the control mix. Almost all the remaining 8 mixes which were made with 10% silica fume, showed high compressive strengths as compared to mixes made with only OPC as the binder. In general, among all 18 mixes, the highest compressive strength after 28 days was obtained for SF-10/P50-1 and the lowest strength for OPC/TW30-3. In addition, an increase in the strength of specimens was also observed. The mixes that contained tea waste and perlite particles contributed higher strengths at 28 days as compared to the mixes with these materials at 7 days. The gain in strength at later ages might be due to the release of all internally cured water from ICAs to contribute to longer hydration and resulting in the formation of more calcium-silicate-hydrate (C-S-H) gel to densify the microstructure.

The results obtained for the compressive strength of the specimens were likened with the reported values found in literature. Sakulich and Bentz [49] strained to mitigate autogenous shrinkage in alkali activated slag (AAS) mortars by internal curing with lightweight aggregates. They found a reduction in compressive strengths by the internal curing method at early ages. They found that such a reduction in strength might be due to either the reduced strength of LWAs or the weak bond strength between cement paste and LWAs. Dybeł and Furtak [50] partially replaced OPC with silica fume to study the influence of silica fume in high-performance concretes. A replacement up to 10% was found to be appropriate for gaining high compressive strengths up to 100 MPa after normal curing of 28 days. Liu et al. [46] reported a reduction in strength of cenosphere-containing mortars at the early ages compared to control mixes. Jakhrani et al. [26] used tea waste and perlite particles in high-strength mortars to control early age hydration and heat release rates. They found a reduction in compressive strengths as compared to the control mix after curing for 3 days. Kim et al. [51] made high-volume fly ash (HVFA) cement mortars with slag as fine aggregate with a 0.45 w/b ratio. They attained higher compressive strengths than the control mix after 28 days. Ahmed et al. [52] added animal hair as reinforcement to the concrete, and obtained higher compressive strengths at fiber amounts up to 0.375%. Further addition of such fibers resulted in decreased strength. The compressive strength outcomes from this research study are found consistent with the results reported in the literature. From the evaluation of compressive strength test results, it is established that the addition of tea waste particles reduces the compressive strength of specimens, however, a gain in strength increases with the passage of time. More gain is found in the specimens that contained tea waste and perlite particles with the addition of silica fume [26].

### 3.4. Flexural Strength

**Figure 5:**
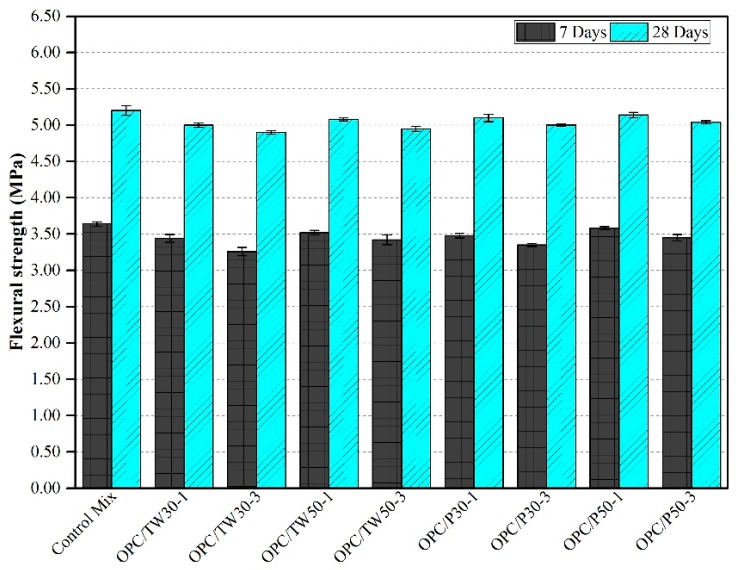
Flexural strength of mortars made with tea waste and perlite particles with 100% OPC.

**Figure 6:**
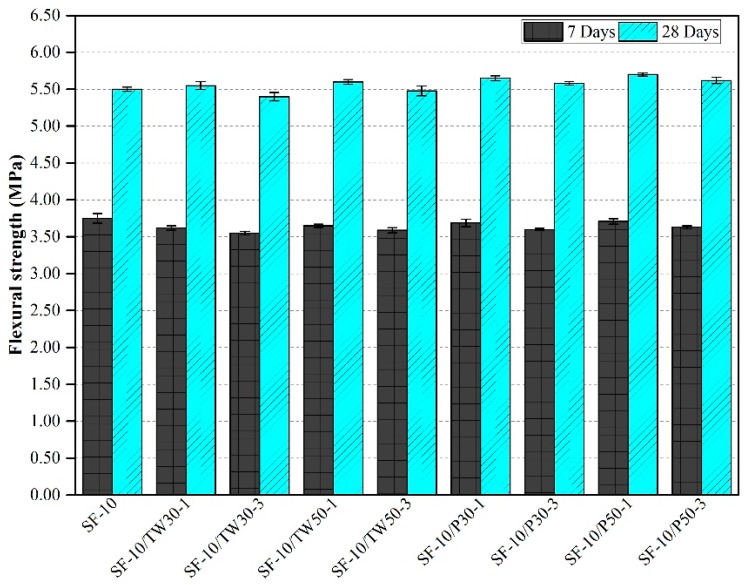
Flexural strength of mortars made with tea waste and perlite particles with 10% SF.

The consequences obtained for flexural strength in cast mixes are shown in Figure 5 and Figure 6. Figure 5 shows the strengths of the first nine mixes that were made with only OPC as a binder. The average flexural strength of the control mix after 7 and 28 days was found as 3.64 MPa and 5.20 MPa, respectively; whereas, the flexural strength of other eight specimens was found to be slightly lesser than the control mix. Figure 6 indicates the strengths of the remaining nine mixes that were made with partial substitution of SF. The addition of SF results in enhanced flexural strengths than without silica fume. The mix SF-10 gave the strength values of 3.75 and 5.50 MPa after 7 and 28 days, respectively. Among the remaining eight mixes, high strengths were obtained in most of the mixes. The strengths of specimens were found higher than the mix SF-10 and those made with only OPC as a binder. Overall, among all 18 mixes, the highest flexural strength was obtained for mix SF-10/P50-1, which was 5.70 MPa after 28 days, and the lowest was from mix OPC/TW30-3 with 4.90 MPa after 28 days. Moreover, the mixes that contained tea waste and perlite particles contributed higher strengths at 28 days as compared to 7 days. As discussed in the previous section, the later gain in strength might be due to the release of all internally cured water from the pores of ICAs to deliver longer hydration and thus resulting in the formation of more C-S-H gel. 

The acquired outcomes of flexural strength were also compared with the results reported in literature. Jakhrani et al. [26] used tea waste and perlite particles as internal curing agents in high-strength cement-based mortars, and found a reduction in flexural strengths after 3 days of curing. Huang et al. [53] used tea waste powder in hemi hydrate gypsums, and found improvement in the strengths by adding a very small amount. Further addition beyond that limit led to a reduction in strengths. Liu et al. [46] used cenospheres as internal curing agents to control autogenous shrinkage. Reduction in the strength for mortars was achieved with the addition of perforated cenospheres as compared to the control mix. Jiang et al. [54] used different nano and micro-fillers in the concrete. The flexural strength results from this study are found consistent with the results reported in the literature. It can be seen that increasing amount of tea waste reduces the strength without the addition of silica fume. Moreover, the addition of silica fume improves the strength in the specimens [55,56], which contained tea waste and perlite particles as compared to those specimens without SF [26].

### 3.5. Ultrasonic Pulse (UP) Velocity

**Figure 7:**
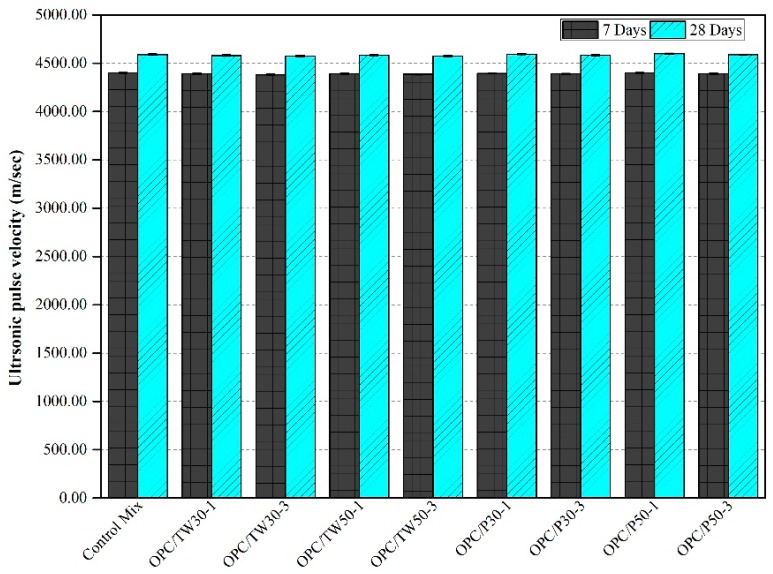
Ultrasonic pulse velocity of mortars made with tea waste and perlite particles with 100% OPC.

**Figure 8:**
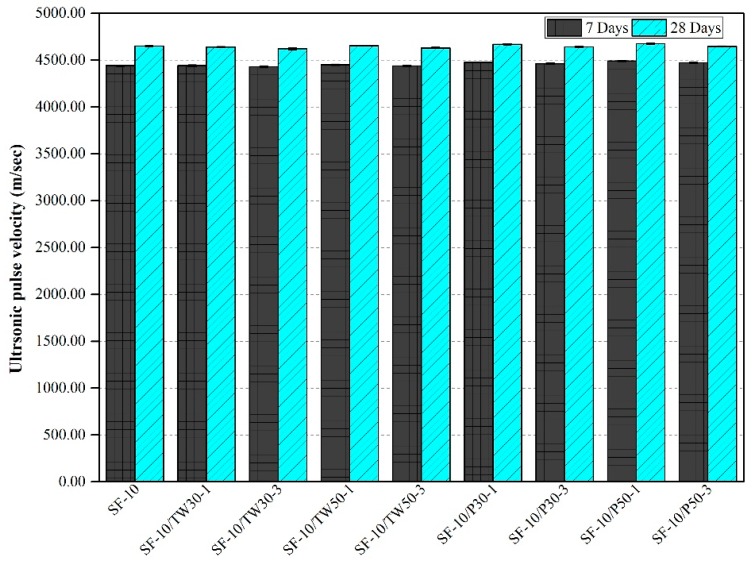
Ultrasonic pulse velocity of mortars made with tea waste and perlite particles with 10% SF.

The ultrasonic pulse velocities of all mix specimens were measured, and their average obtained values are shown in Figure 7 and Figure 8. Figure 7 demonstrates UP velocities of the first nine mixes, which were made with only OPC as a binder. The average UP velocities of the control mix after 7 and 28 days were found to be 4400 and 4590 m/sec, respectively. The UP velocities of the other eight mixes were slightly lower than that of the control mix except OPC/P30-1 and OPC/P50-1, which showed the average UP velocities of 4394 and 4594 m/sec and 4400 and 4598 m/sec after 7 and 28 days curing time, respectively. Figure 8 illustrates the strengths of the remaining nine mixes that were made with silica fume as a secondary binder. It was found that the addition of SF results in higher velocities of specimens than the mixes which are without silica fume. The mix SF-10 gave the velocities of 4440 and 4650 m/sec after 7 and 28 days, respectively. Among the remaining eight mixes, high velocity values were obtained in three mixes only, which were SF-10/P50-1, SF-10/P30-1 and SF-10/TW50-1. In general, among all 18 mixes, the highest UP velocity value was observed for the mix SF-10/P50-1 that had a velocity of 4675 m/sec after 28 days, and the lowest UP velocity value of 4575 m/sec was achieved for the mix OPC/TW30-3 after 28 days of curing. 

The ultrasonic pulse velocity results of the research work are similar to that of previous studies. Jakhrani et al. [26] used tea waste and perlite particles in their study to find the effect on hydration of high-strength mortars at early ages. They found that the addition of tea waste and perlite particles have a contrary influence on UP velocities. It was suggested that such results might be due to the porous nature of used materials. The reduced UP velocity values were observed in the specimens that were made with TW and perlite particles without SF. The reduction in UP velocities is attributed to the presence of porous tea waste and perlite particles in the respective specimens. However, the porosity may disappear after a long time by making denser microstructure because of the existence of additional water in the pores for complete hydration.

### 3.6. Densities and Absorption Rate of Hardened Mortar Specimens

**Figure 9:**
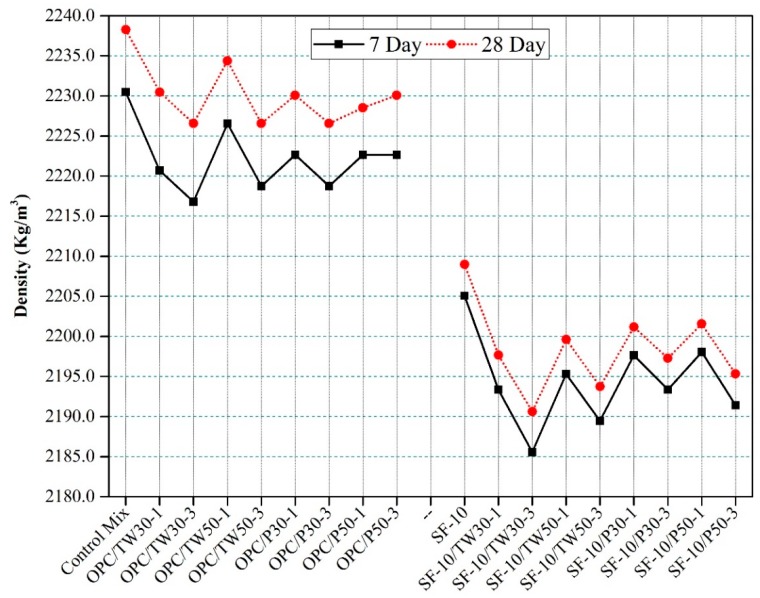
Densities of specimens made with tea waste and perlite particles with OPC and SF as binders.

**Figure 10:**
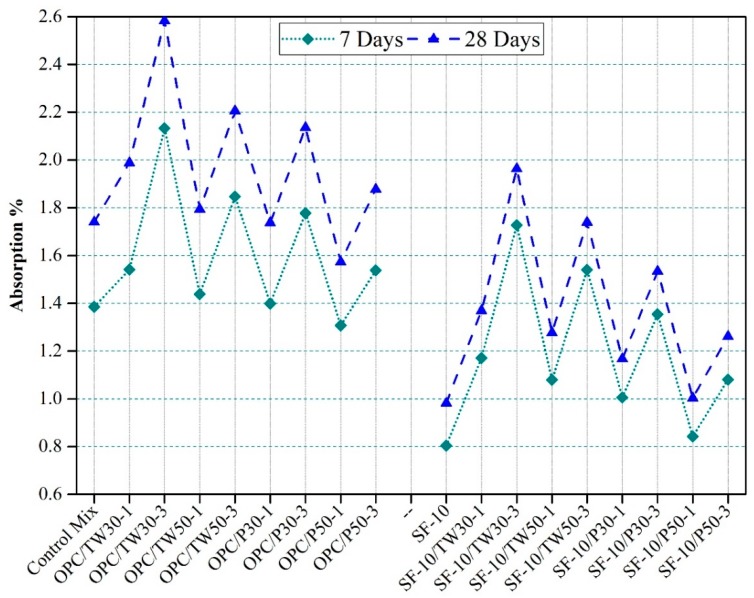
Densities of specimens made with tea waste and perlite particles with OPC and SF as binders.

Figure 9 shows the densities of prismatic mortar specimens of all 18 mixes. The 7 and 28 daya densities of the control mix were high among all mixes. The densities of other specimens containing tea waste particles were found to be reducing with the increasing amount of tea waste and perlite particles. The specimens made with a partial addition of silica fume also showed lower density values as compared to the mixes made with OPC as the only binder. Moreover, the 7 days densities were lower than 28 days densities. This might be due to the absorption of water by particles to increase densities. Figure 10 represents the absorption rate of all 18 mixes. The specimens containing tea waste particles showed higher absorption rates than other mixes. More absorption was observed in mixes that contained coarser particles with OPC as the only binder. However, the addition of silica slightly reduced the absorption rate. 

Many authors found the phenomenon behind the reduction of densities in high-strength mortars and concretes. Al Saffar et al. [8] and Mechtcherine et al. [38] recognized the reduction in densities of specimens that contained internal curing agents and deduced that the reduction might be due to the lower densities of used ingredients and ICAs. However, in some studies, an increase in densities was observed at later ages. Gesog˘lu et al. [40] used LWAs by replacing normal-weight fine aggregates (NWFAs) with different replacement levels. They found lower density values, and reported that this might be due to lower densities of LWAs. In addition, Rashad [22] conducted a review on usage of different types of materials used in concretes. In his studied literature, he established that the addition of expanded perlite in the cement matrix increased the absorption capabilities and porosity of matrix.

Our study shows similar tendencies with respect to the studied literature. The lower densities of specimens containing tea waste and perlite particles were attributed to their low densities. 

### 3.7. Scanning Electron Microscopy

**Figure 11:**
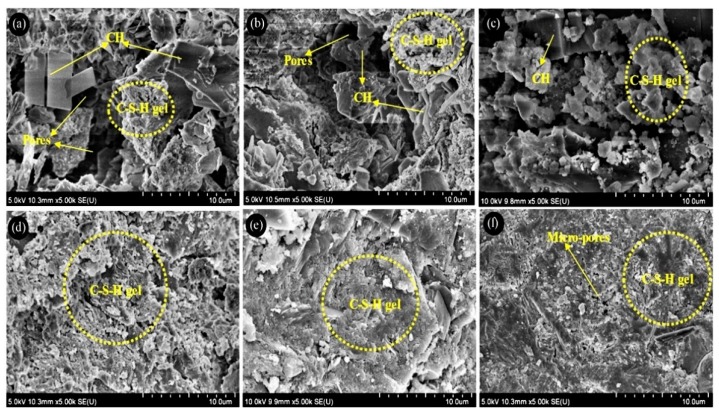
SEM images of mortar specimens cured for 28 days. (**a**) Control mix, (**b**) OPC/TW50-1, (**c**) OPC/P50-1, (**d**) SF-10, (**e**) SF-10/TW50-1 and (**f**) SF-10/P50-1.

SEM images of only six mixes (control mix, OPC/TW50-1, OPC/P50-1, SF-10, SF-10/TW50-1 and SF-10/P50-1) after curing for 28 days were analyzed as shown in Figure 11. Major hydration products, such as portlandite (CH) and calcium-silicate-hydrate (C-S-H) gel, were seen during the SEM evaluation. Figure 11a represents the SEM image of the control mix. Presence of portlandite and C-S-H gel can be witnessed in the control mix and it contains larger pores as compared to the other mixes. Hence, that might be a reason for lower strengths in the case of the control mix. Figure 11b,c shows the SEM images of mixes OPC/TW50-1 and OPC/P50-1. A denser structure can be witnessed in this case, which might be due to complete hydration because of internal curing and thus resulting in a denser structure and more C-S-H gels as compared to the control mix. Moreover, the OPC was partially replaced with 10% silica fume in Figure 11d,e. Figure 11d shows the mix SF-10, which is comprised of 10% silica fume without tea waste and perlite particles. It can be seen that the addition of silica fume densified the microstructure of paste as compared to the control mix and portlandite was witnessed at 28 days. However, Figure 11e,f shows the addition of tea waste and perlite particles with silica fume. It can be seen that a cleaner and denser structure is formed as compared to the mix specimen made with only OPC and SF-10. Hence, the denser microstructures are attributed to the presence of internal curing agents for further hydration to make more C-S-H to get higher strengths.

Silica fume is an excellent pozzolanic material which reacts with calcium hydroxide, resulting in the formation of calcium silicate hydrate at early ages [57]. Justs et al. [43] used SAPs to make ultrahigh performance concrete (UHPC). They conducted SEM analysis to investigate the microstructure of paste and observed that the added SAP reservoirs were partially filled with hydration products. In our case, the specimens with tea waste and perlite particles demonstrated an improved microstructure with silica fume as compared to specimens made with only OPC. This is because the water present inside the tea waste and perlite particles released slowly and contributed to the extended hydration of the cement.

### 3.8. Thermo-Gravimetric Analysis

**Figure 12:**
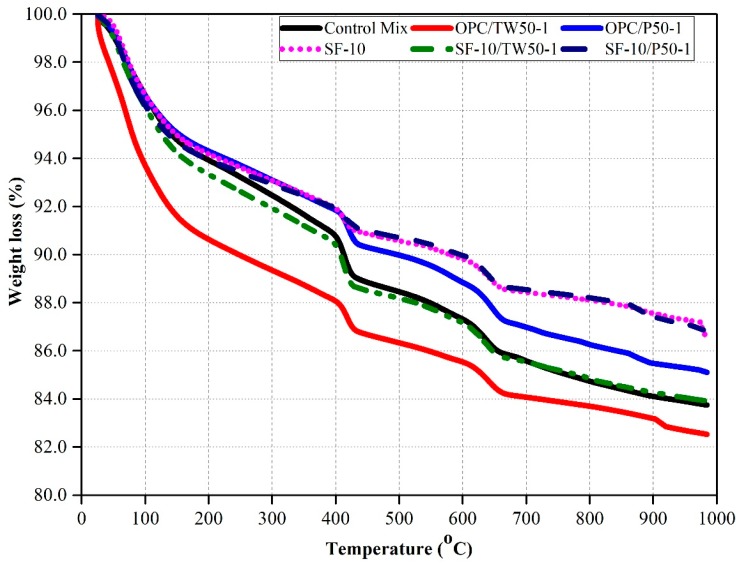
Thermo-gravimetric analysis (TGA) of samples made with tea waste and perlite particles with and without SF after 28 days.

Perkin Elmer STA8000 instrument was used to analyze the weight loss of six mix specimens. The TGA results of specimens are shown in Figure 12. In the case of all six mixes (control mix, OPC/TW50-1, OPC/P50-1, SF-10, SF-10/TW50-1 and SF-10/P50-1), total weight loss of about 16.0%, 18.0%, 15.0%, 13.0%, 16.0% and 13.0%, were recorded at 1000 °C. More weight loss was attained for mix OPC/TW50-1, and very low weight loss was observed for mixes OPC/P50-1 and SF-10. In the case of mix OPC/TW50-1, 6% weight loss was achieved at 100 °C, 8% at about 101–600 °C and 4% at about 601–1000 °C. The results reveal that the specimens that contained tea waste particles show more weight loss as compared to other mixes, and more weight loss was observed at temperature ranges between 101 and 600 °C.

The weight loss at 0–100 °C [58], 101–600 °C and 601–1000 °C is due to the evaporation of physical water [31,58], burning of organic matter and evaporation of chemically-bound water, and de-hydroxylation of oxides and chlorites [31], respectively. Low weight loss occurred in mixes that contained silica fume, which might be due to low available water due to complete and enhanced hydration.

## 4. Conclusions

The aim of this research work was to investigate the effect of tea waste particles on prevention of autogenous shrinkage in high-strength mortars at early ages and its effect on other parameters of high-strength mortars. Therefore, the use of black tea waste was considered in this research for minimizing its adverse environmental impact generated by its disposal. The following major findings were obtained during this research study.

- The use of an increasing amount of internal curing agents (black tea waste and perlite) particles with OPC as the only binder, resulted in:
  ○ Lower flow ability of fresh mortar as compared to the control mix. Tea waste particles reduced flow more as compared to perlite particles. In addition, the coarser particles showed a greater reduction in flow values than finer particles;
  ○ Reduced autogenous shrinkage as compared to the control mix. A greater reduction in shrinkage was observed in the mixes that contained tea waste particles. Similar to flow values, coarser particles showed more reduction in shrinkage as compared to finer particles;
  ○ Low compressive and flexural strength values were obtained. Reduction in strength was observed in the specimens that contained tea waste and perlite particles; however, strength gain ratio was found to increase at later ages. The coarser particles showed lower strength values than finer particles;
  ○ Similar to strength values, low ultrasonic pulse velocity values were obtained. A greater reduction was seen in the specimens that contained tea waste particles as compared to perlite particles;
  ○ Low density and high absorption rates were obtained in the mixes that contained tea waste and perlite particles as compared to the control mix. The use of silica fume also showed lower density and absorption values as compared to mixes that were made with OPC as the only binder. The lower densities in mixes were attributed to lesser densities of used ingredients; however, the high absorption rates were attributed to the porous structure of used materials;
  ○ A porous microstructure was seen through SEM analysis in the control mix as compared to mixes that contained tea waste and perlite particles at 28 days. Although the same effect can be seen through the strength analysis, where gain in strength was increasing;
  ○ More weight loss was found in mixes that contained tea waste particles as compared to the control mix and mixes made with perlite particles. The loss in weight might be due to the evaporation of evaporable water that was inside the pores and the burning of tea waste particles.
- The use of black tea waste and perlite particles with partial addition of silica fume, resulted in:
  ○ Increased flow and autogenous shrinkage of mortars as compared to specimens without silica fume. High flow ability might be due to round particle shapes of silica fume, and the high shrinkage values might be because of the quick reaction of silica fume particles in presence of water;
  ○ Increased compressive and flexural strengths and ultrasonic pulse velocity values were obtained as compared to the control mix and other respective mixes. It might be because of the quick reaction of silica fume particles to gain strength at early ages;
  ○ Densified microstructure and reduced weight loss at age of 28 days. The denser structure in the presence of silica fume was due to filling micro-pores with silica fume particles and the formation of more C-S-H gel in pores of composite, which thus resulted in a reduction in weight loss.

## 5. Future Work Under Consideration 

We plan to determine the potential usage of tea waste particles as a carrier for phase change materials (PCMs) for self-healing and the durability of high-strength mortars under sulfate and acid exposures.

## Figures and Tables

**Table 1:** Chemical composition and physical properties of used binders.

Description	Results	
Chemical Composition of Binders (wt. %)	Ordinary Portland Cement (OPC)	Silica Fume (SF)
CaO	62.00	1.50
SiO_2_	20.80	88.70
Al_2_O_3_	6.30	1.80
Fe_2_O_3_	3.20	1.80
MgO	3.30	0.80
SO_3_	2.20	0.10
Na_2_O	0.60	0.33
LOI	1.30	1.10
**Physical Properties**		
Specific gravity (g/cm^3^)	3.15	2.20
Blaine fineness (m^2^/kg)	325.00	20,000.00

**Table 2:** Physical properties of two different used types of tea waste and perlite particles.

Description	Results			
Physical Properties	TW30	TW50	P30	P50
Specific gravity (g/cm^3^)	0.342	0.465	0.345	0.650
BJH adsorption cumulative surface area (cm^2^/g)	1.307	1.773	0.832	1.107
BJH adsorption cumulative pore volume (cm^3^/g)	0.000819	0.001055	0.00694	0.00376
BJH adsorption pore diameter (Å)	25.066	23.809	33.70	13.582
Absorption (%)	215.00	110.00	75.00	47.00
Moisture content (%)	3.50	2.10	2.10	1.50

TW: Tea waste; P: Perlite; BJH: Barrett-Joyner-Halenda Analysis.

**Table 3:** Elemental composition of used black tea waste particles.

Description	Results
Elemental Composition	Tea Waste
Carbon (C) (mass %)	49.34
Oxygen (O) (mass %)	39.60
Nitrogen (N) (mass %)	07.89
Calcium (Ca) (Mass %)	01.31
Phosphorous (P) (ppm)	3802.00
Sulphur (S) (ppm)	3443.00
Potassium (K) (ppm)	2829.00
Silicon (Si) ppm	2020.00
Iron (Fe) (ppm)	1564.00
Aluminum (Al) (ppm)	1405.00

**Table 4:** Detailed mix proportion.

Mix ID	OPC by Weight	SF Ratio by Weight of Cement	Binder/Sand (B/S) Ratio	TW30 % by Volume of Cement	TW50 % by Volume of Cement	P30 % by Volume of Cement	P50 % by Volume of Cement
Control mix	1.0	−	1.25	−	−	−	−
OPC/TW30-1	1.0	−		1.0	−	−	−
OPC/TW30-3	1.0	−		3.0	−	−	−
OPC/TW50-1	1.0	−		−	1.0	−	−
OPC/TW50-3	1.0	−		−	3.0	−	−
OPC/P30-1	1.0	−		−	−	1.0	−
OPC/P30-3	1.0	−		−	−	3.0	−
OPC/P50-1	1.0	−		−	−	−	1.0
OPC/P50-3	1.0	−		−	−	−	3.0
SF-10	0.9	0.1		−	−	−	−
SF-10/TW30-1	0.9	0.1		1.0	−	−	−
SF-10/ TW30-3	0.9	0.1		3.0	−	−	−
SF-10/ TW50-1	0.9	0.1		−	1.0	−	−
SF-10/ TW50-3	0.9	0.1		−	3.0	−	−
SF-10/P30-1	0.9	0.1		−	−	1.0	−
SF-10/ P30-3	0.9	0.1		−	−	3.0	−
SF-10/ P50-1	0.9	0.1		−	−	−	1.0
SF-10/ P50-3	0.9	0.1		−	−	−	3.0
